# Progressive Thymic Hyperplasia With Graves’ Disease: A Case Report

**DOI:** 10.7759/cureus.43950

**Published:** 2023-08-22

**Authors:** Noriko Ogawa, Miwako Yomota, Masakazu Notsu, Keizo Kanasaki

**Affiliations:** 1 Division of Endocrinology and Metabolism, Shimane University Faculty of Medicine, Izumo, JPN

**Keywords:** thiamazole, thyroidectomy, sil-2r, thymic hyperplasia, graves’ disease

## Abstract

We treated a 36-year-old man with thymic hyperplasia complicated with Graves’ disease. Thymic hyperplasia was observed on thoracic computed tomography (CT) three months after the onset of thyrotoxicosis symptoms. One month after thiamazole initiation, he displayed drug-induced liver injury and underwent a total thyroidectomy. Seven months after surgery, we observed a dramatic reduction of thymic size associated with normalizing the soluble interleukin-2 receptor (sIL-2R) levels. The rapid development of hyperplasia after the onset of thyrotoxicosis and the restoration in thymus volume after total thyroidectomy associated with suppression of sIL-2R, in this case, suggests the complexity of the pathogenesis of thymic hyperplasia in the thyrotoxicosis.

## Introduction

The thymus is a primary lymphoid organ that lies in the superior mediastinum and may extend into the neck. Thymic hyperplasia associated with Graves’ disease has been reported [1]. The thymic hyperplasia can be cured with Graves’ disease treatment; therefore it does not require specific treatment [2], even though some cases were diagnosed as hyperplasia after thymectomy. Possible causes of thymic hyperplasia in Graves’ disease include the direct effect of elevated thyroid hormones on the thymus [3], stimulation of the thymus with thyroid stimulating hormone (TSH) receptor antibodies [4,5], and immune abnormalities associated with Graves’ disease [6,7]. Here we present a case with gross thymic mass lesion initially planned for thymectomy for substantial doubt as thymic cancer due to rapid thymic enlargement with weight loss of approximately 10 kg. However, his thymic lesion was improved with a thyroidectomy of Graves’ disease.

## Case presentation

**Figure 1:**
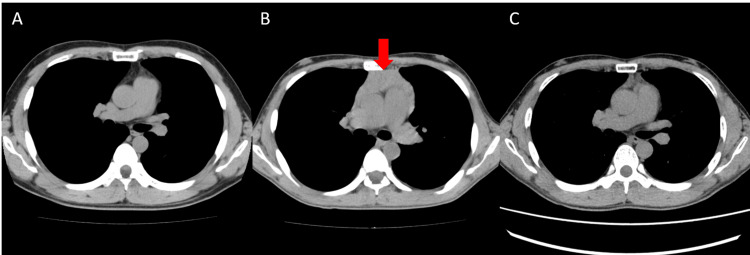
Changes over time in the thymic hyperplasia on chest CT scans A: Two months before the onset of thyrotoxicosis symptoms. B: Three months after the onset of thyrotoxicosis symptoms, the thymic was enlarged. C: Seven months after surgery, the thymic hyperplasia had decreased on chest CT. CT: computed tomography

**Figure 2:**
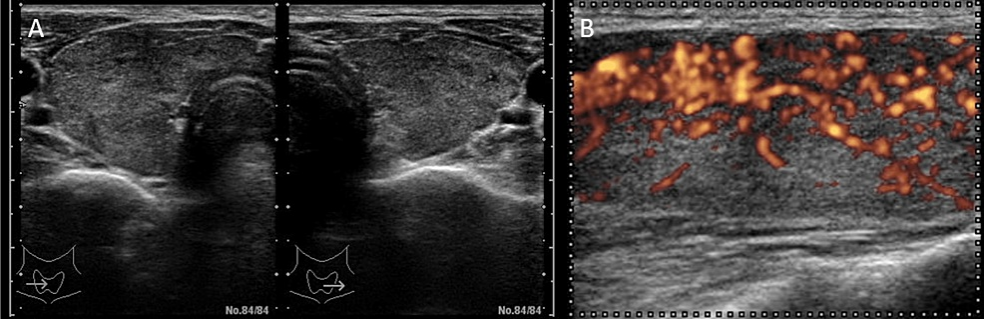
Diffuse goiter on ultrasonic examination A: Diffuse goiter. B: Thyroid gland with increased blood flow.

**Figure 3:**
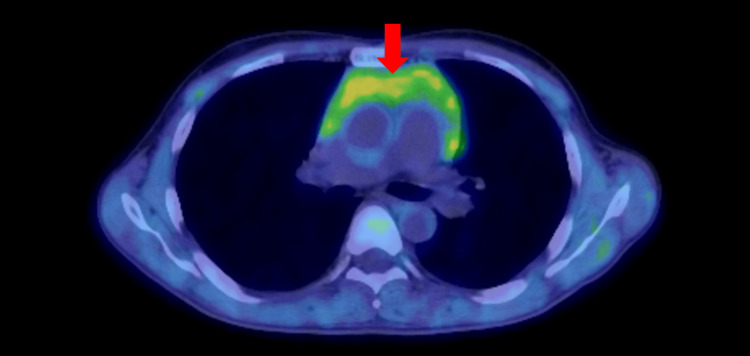
Positron emission tomography-computed tomography in the patient

A 36-year-old man visited our clinic due to thyrotoxicosis. Two years before this evaluation, multiple granular shadows were noted on his chest X-ray photography during a health checkup two years ago, and he had followed up by chest computed tomography (CT) every six months at a local doctor. Six months ago, the chest CT showed no abnormalities in the thymus (Figure 1A). In the past four months before admission, he had suffered fatigue, palpitations, hand tremors, anorexia, thirst, and hyperidrosis. His weight lost nearly 10 kg in three months. Last month, a chest CT revealed that he had an enlargement of the thymus (60 × 88 × 28 mm) (Figure 1B). Since his thymus size rapidly enlarged, a total thymectomy on thoracic surgery was planned. He was 165.6 cm tall, weighed 53.9 kg, and had a blood pressure of 139/77 mmHg and a pulse of 107/min. The diffuse goiter was palpable. He had no exophthalmos. Superficial lymph nodes in the neck and supraclavicular region were not palpable. Conjunctiva was without anemia or jaundice. No abnormality was in cardiopulmonary sounds. General blood examination results showed no abnormalities. Endocrinological and immunological examinations revealed an elevation of FT3 and FT4 with TSH suppression. Both thyrotropin receptor antibody (TRAb) and TsAb were positive (TSH was 0.605 μU/mL, free T4 was 6.1 ng/dL, free T3 was >25.0 pg/mL, TRAb was 11.8 IU/L, TsAb was 2260%). The thyroid gland was diffusely enlarged (Figure 2A) with increased vascular flow on ultrasonography (Figure 2B). These findings were consistent with Graves’ disease. Thymic enlargement showed internal homogeneity on CT and no abnormal accumulation on positron emission tomography CT (PET-CT) (Figure 3). Tumor markers suspicious for thymic malignancy were not increased (carcinoembryonic antigen (CEA) was 0.8 ng/mL, alpha-fetoprotein (AFP) was 4.5 ng/mL, and neuron-specific enolase (NSE) was 12.1 ng/mL). To treat Graves' disease, an antithyroid drug, thiamazole 15 mg daily, was initiated. One month later, FT4 level was decreased to 2.9 ng/dL, whereas white stools and liver injury were observed (total bilirubin (T-Bil) was 3.1 mg/dL, aspartate aminotransferase (AST) was 111 U/L, alanine aminotransferase (ALT) was 284 U/L, alkaline phosphatase (ALP) was 841 U/L, gamma-glutamyl transferase (γ-GTP) was 377 U/L). Viral hepatitis was negative (HBsAg, HBcAb, and HCVab were negative), and γ-globulins were not increased, suggesting drug-induced liver injury. Thiamazole was ceased, and 100 mg potassium iodide was initiated instead. One month later, a total thyroidectomy was performed. The thyroid function has remained within the normal range on 100 µg levothyroxine replacement therapy. TRAb levels decreased to 3.4 IU/L four months after surgery and subsequently normalized. Seven months after surgery, soluble interleukin-2 receptor (sIL-2R) levels had decreased to 323 U/mL within the normal range from 2306, and thymic hyperplasia had diminished on chest CT (Figure 1C).

## Discussion

Here, we presented a case of regression of thymic hyperplasia following curative thyroidectomy for Graves’ disease. It is imperative to perform differential diagnosis for thymoma, which is treated as a malignant tumor to thymic hyperplasia. MRI is helpful in this assessment of internal homogeneity and decreased chemical shift signal intensity in the thymus [8]. We diagnosed and took a wait-and-see approach as a thymic hyperplasia because the enlarged thymus showed internal homogeneity on CT and no abnormal accumulation on PET-CT, no increase in tumor markers CEA, AFP or NSE suspicious for thymic malignancy, and thyrotoxicosis was observed, despite MRI was not performed in the present case.

Thymic hyperplasia complicating Graves’ disease has been reported. Thymic hyperplasia is morphologically classified into true thymic hyperplasia and lymphofollicular thymic hyperplasia, associated with Graves’ disease [9]. The lymphofollicular hyperplasia occurs in 1-2% of healthy individuals [10,11] and is also found in other autoimmune diseases such as myasthenia gravis, SLE, systemic scleroderma, rheumatoid arthritis, and Sjögren's syndrome, also liver cirrhosis and diabetes mellitus [12]. In a study of thymic biopsies in patients undergoing thyroidectomy for thyrotoxicosis, Gunn and Michie et al. reported a 32-38% incidence of thymic hyperplasia in patients with thyrotoxicosis [13,14]. Simpton et al. described thymic hyperplasia in all 158 thyrotoxicosis patients for whom thymic biopsies were performed [15]. These findings suggest that thymic hyperplasia is very common at a histological level in patients with thyrotoxicosis.

The clear pathogenesis of thymic hyperplasia associated with Graves' disease is not elucidated yet, though thyrotoxicosis would play a role. The thymectomy in patients with Graves’ disease did not affect hyperthyroidism [16]. In addition, the enlargement of thymus volume was observed with worsening of thyroid function; the restoration of thyroid function reduced in thymus size [17]. Thyroxine administration causes a thymic enlargement in mice and laying hens [18,19], and the thyroid hormones stimulate thymic epithelial proliferation in vitro [3]. On the contrary, thyroidectomy in rabbits hastened thymic involution [20]. Thymic epithelial cells express nuclear T3 receptors, which may stimulate thymocyte differentiation [21], and thyroid hormones have been shown to lead to T-cell proliferation and differentiation via the secretion of a thymic factor called thymulin [22]. In the present case, thymic hyperplasia was observed after the onset of thyrotoxicosis; total thyroidectomy-induced cure in thyrotoxicosis restores the thymus size, supporting the role of thyroid hormone in the pathogenesis of thymus hyperplasia.

The negative conversion of TRAb coincided with a period of improved thymic hyperplasia in this case. It has also been presented that immunoglobulins from patients with Graves’ disease induce a binding affinity and mitogenic response to thymocytes in vitro [23]. The functional TSH receptors, activated by TSH binding, are expressed in thymocytes, suggesting that stimulation with TSH receptor antibodies may lead to thymic hyperplasia [4,5]. However, the contribution of TRAb to thymic hyperplasia is controversial. Some reports described that TRAb is involved in thymic hyperplasia; however, a case of thymic hyperplasia had shrunk during the remission of Graves’ disease despite persistently positive TRAb levels [17]. Jinguji M demonstrated that the thymic volume decline rate positively correlated with serum T3 but not with TRAb levels [24].

Furthermore, the role of elevated TSH levels in hypothyroidism on thymus hyperplasia would be questionable. Only two cases of thymic hyperplasia with primary hypothyroidism have been reported, a case during levothyroxine supplementation [25] and a case of Hashimoto disease with thymic hyperplasia and splenomegaly after sudden death [26]. The thyroid hormone-induced thymic enlargement has been observed in animals without Graves' disease, and a case of thymic enlargement was reported during the process of thyroid hormone elevation with levothyroxine in primary hypothyroidism [25]. In this case, thymic hyperplasia decreased after normalization of thyroid function following total thyroidectomy, and the negative conversion period of TRAb was consistent with previous reports with total thyroidectomy [27]. The thymic hyperplasia in the present case can be primarily due to improved thyroid function, and the negative conversion of TR-Ab may just be a change associated with total thyroidectomy, with little influence on thymic hyperplasia.

In the present case, serum sIL-2R level was elevated. The sIL-2R increases with hyperthyroidism and decreases with treatment [28]. The sIL-2R could be elevated by hyperthyroidism alone, and thyroid hormone induces T-cell auto-activation [6,7]. As IL2 binds to IL2R to proliferate and differentiate T cells [29], the activation of T cells by thyroid hormones may also induce thymic enlargement. The role of such an IL-2R-mediated system in developing thymus hyperplasia in thyrotoxicosis would require further information.

## Conclusions

Here we described the case of Graves’ disease-associated thymic hyperplasia, which enlarged rapidly after the onset of thyrotoxicosis. We have concluded that thyroid hormones have a significant driver on thymic hyperplasia and that some immune abnormality derived from elevated thyroid hormones may augment on top of such effects, leading to massive thymic hyperplasia.
